# Modeling Timbre Similarity of Short Music Clips

**DOI:** 10.3389/fpsyg.2017.00639

**Published:** 2017-04-26

**Authors:** Kai Siedenburg, Daniel Müllensiefen

**Affiliations:** ^1^Department of Medical Physics and Acoustics, Carl von Ossietzky University of OldenburgOldenburg, Germany; ^2^Department of Psychology, Goldsmiths University of LondonLondon, UK

**Keywords:** short audio clips, music similarity, timbre, audio features, genre

## Abstract

There is evidence from a number of recent studies that most listeners are able to extract information related to song identity, emotion, or genre from music excerpts with durations in the range of tenths of seconds. Because of these very short durations, timbre as a multifaceted auditory attribute appears as a plausible candidate for the type of features that listeners make use of when processing short music excerpts. However, the importance of timbre in listening tasks that involve short excerpts has not yet been demonstrated empirically. Hence, the goal of this study was to develop a method that allows to explore to what degree similarity judgments of short music clips can be modeled with low-level acoustic features related to timbre. We utilized the similarity data from two large samples of participants: Sample I was obtained via an online survey, used 16 clips of 400 ms length, and contained responses of 137,339 participants. Sample II was collected in a lab environment, used 16 clips of 800 ms length, and contained responses from 648 participants. Our model used two sets of audio features which included commonly used timbre descriptors and the well-known Mel-frequency cepstral coefficients as well as their temporal derivates. In order to predict pairwise similarities, the resulting distances between clips in terms of their audio features were used as predictor variables with partial least-squares regression. We found that a sparse selection of three to seven features from both descriptor sets—mainly encoding the coarse shape of the spectrum as well as spectrotemporal variability—best predicted similarities across the two sets of sounds. Notably, the inclusion of non-acoustic predictors of musical genre and record release date allowed much better generalization performance and explained up to 50% of shared variance (*R*^2^) between observations and model predictions. Overall, the results of this study empirically demonstrate that both acoustic features related to timbre as well as higher level categorical features such as musical genre play a major role in the perception of short music clips.

## 1. Introduction

There is growing evidence that human listeners are able to instantly categorize short music clips containing complex mixtures of sounds, e.g., when scanning a radio dial or browsing through a playlist. Even more, the information contained in clips lasting only a few hundred milliseconds or less seems to be sufficient to perform tasks such as genre classification (Gjerdingen and Perrott, 2008; Mace et al., 2011; Plazak and Huron, 2011) or artist and song recognition (Schellenberg et al., 1999; Krumhansl, 2010).

More specifically, Gjerdingen and Perrott (2008) played participants audio excerpts of commercially available music at different lengths and asked them to indicate the genre of each excerpt. They found that 44% of participants' genre classifications of 250 ms excerpts were identical to the classifications by the same participants and of the same audio track when played for 3 s, demonstrating that listeners extract a considerable amount of information from very short excerpts. Results by Schellenberg et al. (1999) showed that even 100 ms excerpts could be matched to song title and artists with above-chance accuracy, and that time- varying high frequency information (>1 kHz) was particularly important for correct identification. Similarly, Krumhansl (2010) showed that listeners are able to identify the artists and titles for 25% of a stimulus set consisting of 400 ms clips of popular music spanning four decades. Mace et al. (2011) were able to demonstrate that even at 125 ms length participants were able to achieve an accuracy of 54% on a genre recognition task which had a guessing level of 20%. At this timescale there are few, if any discernible melodic, rhythmic, harmonic or metric relationships to base judgements on. Though when musical-structural information is minimal, timbral information can still be rich.

Timbre is here understood as an umbrella term that denotes the bundle of auditory features (other than pitch, loudness, duration) that contribute to both sound source categories and sound quality (McAdams, 2013). In fact, timbre seems to be processed even from very short stimulus durations. For instance, Bigand et al. (2011) showed that variability in the spectral envelope can be processed from sounds as short as 50 ms. More recent results by Suied et al. (2014) have shown that listeners can even recognize timbre based on snippets as short as 16 ms (depending on the instrument family). In the latter study, performance increased monotonically with the length of the excerpts and plateaued at around 64 ms.

Building on this research, Musil et al. (2013) devised an individual differences test that investigates differences in the ability to extract information from short audio clips and to use it for similarity comparisons. This test forms part of the Goldsmiths Musical Sophistication battery of listing tests (Müllensiefen et al., 2014) and complements other individual differences tests that focus on melodic memory and beat perception abilities. The sound similarity test was designed to assess the ability to decode and compare complex musical sound textures and to be independent of temporal processing and memory capabilities and therefore only makes use of very short musical stimuli. While the test has been used in practice and proved to be fairly unrelated to other musical listening abilities (Müllensiefen et al., 2015), it has been difficult to build a model based on audio features that would describe participants' similarity judgements adequately (Musil et al., 2013).

On the contrary, there is a rich literature on audio features associated with computer-based instrument identification (Joder et al., 2009), genre classification (e.g., Andén and Mallat, 2011), the prediction of affective qualities (Laurier et al., 2009; McAdams et al., 2017), or more general aspects of the perception of audio excerpts (Alluri and Toiviainen, 2010). Audio features are most commonly derived from the Short-Time Fourier Transform of the music signal, from which spectral or temporal statistics are computed. A standard example are summary statistics such as the mean (i.e., centroid) or spread of short-time spectra, or the correlation of spectra across consecutive time windows (spectral *flux*). It is important to note that the utility of specific timbre descriptors as well as the size of feature sets varies considerably across computational and perceptual tasks. In effect, timbre description in psychology traditionally employs a handful of, say, less than 10 features, whereas many music information retrieval approaches rely on audio representations with a substantially higher dimensionality (Siedenburg et al., 2016a).

None of the psychological studies on short audio clips has used audio features to quantitatively model human perceptual responses to very short audio clips. For that reason, it is currently unclear to which extent simple categorization judgements can be predicted by low-level properties of the audio signal, as opposed to higher level concepts such as genre potentially inferred from the audio. But constructing a cognitively adequate model of audio similarity is not only useful for understanding what features and cues listeners extract and process from short audio clips. It can also serve as a first step for constructing future adaptive versions of individual differences tests of audio classifications that could allow a systematic scaling of difficulty of sets of audio clips by selecting clips that are more or less similar.

This paper aims to contribute toward the understanding of perceptual judgements of similarity for short music clips via a modeling approach. The present contribution is the first study to systematically quantify the extent to which similarity data of short musical excerpts can be explained by acoustic timbre descriptors. A notable feature of the current approach is that we not only evaluate the constructed statistical models in terms of their accuracy in describing a given set of observations, but also in their capacities to generalize to unseen data sets. The predictive accuracy of low-level timbre features is further compared with variables that encode meta information in the form of the genre and release date of songs.

This manuscript is organized as follows. In Section 2, we describe the experimental samples, stimuli, and procedures that provide the basis for our modeling study. In Section 3, the structure of the model is described in detail, in particular with regards to the audio features, normalization schemes, and statistical models of perceptual similarity. In Section 4, the presented models are comprehensively evaluated, before potential implications on timbre modeling are discussed in Section 5.

## 2. Experiments

This study uses data from two separate experiments that used a sorting paradigm to assess the perceptual similarity of short music clips. In both cases the sorting paradigm was part of a larger test battery on several aspects of music perception (Müllensiefen et al., 2014). Only the data gathered via the similarity sorting paradigm is reported in this paper and has not been reported previously. The Ethics Board of Goldsmiths, University of London approved the research undertaken and reported in the manuscript.

### 2.1. Participants

Sample I comprised responses from 137,339 participants who took part in the BBC Lab UK's online test *How Musical Are You?* in 2011 and 2012. The sample of participants is identical to the sample reported by Müllensiefen et al. (2014, Study 4), although the data from the sound similarity test has not been reported previously. In the training sample, 45.2% of the participants were female and mean age was 35.2 years (SD = 15). Participants were mainly UK residents (66.9%) but because the *How Musical Are You?* test was an open online application, the sample also included participants from other mainly Western and English-speaking countries (largest proportions: USA: 14.2%, Canada: 2.3%, Australia: 1.1%). The sample contained a large spread in terms of education (undergraduate degree/professional qualification: 34.1%, still in education: 23.4%, postgraduate degree: 19%, second school degree with around 18 years (e.g., British A-levels): 11.8%, first school degree around 16 years (e.g., British GCSE/O-levels): 7.5%, etc.) as well as in terms of the current profession of the participants (Other: 19.4%, Education/Training: 12.4%, Unemployed: 10.7%, Information technology: 7.1%, etc.). Only 1.8% stated “music” as their occupation. Participants in Sample I were tested with 400 ms excerpts.

Sample II comprised responses from 648 participants, collected via several experimental batteries that were run at Goldsmiths University of London between 2011 and 2014, all of which contained the sound similarity test using 800 ms excerpts. Participants came from a young student population (undergraduates as well as postgraduates) and were less diverse in terms of their educational and occupational background than participants in Sample I^1^.

### 2.2. Stimuli

Prototypical but less well-known songs from four different genres were selected as experimental stimuli, as described by Musil et al. (2013). Because genre boundaries may be subjective and change over time (Gjerdingen and Perrott, 2008), we used the main four meta-genres identified by Rentfrow and Gosling (2003) as a guidance and selected the most prominent popular music style within each meta-category: jazz from reflective/complex, rock from intense/rebellious, pop from upbeat/conventional, and hip-hop from energetic/rhythmic. Additionally, following Krumhansl's (2010) finding that the approximate recording date of a song can be identified fairly accurately from short excerpts, specific decades were selected for each genre: 1960–70s for jazz, 1970–80s for rock, 1990–2000 for pop and hiphop. Exemplary songs for each of these genres were selected from the suggestions of prototypical songs given on the encyclopedic music datbase allmusic.com. In order to avoid the recognition of specific overly well-known tunes, songs were only selected if they were not present in the all-time top-100 Billboard charts and had never reached the top rank on the UK Billboard charts. However, two of the selected songs (*The Sign, I Wanna Love You Forever*) had reached first and third ranks of the US Hot-100 Billboard charts, respectively. Hence, we cannot rule out the possibility that individual participants might have recognized the songs of individual excerpts. Aiming for representative sound fragments, excerpts from each song were chosen such that the excerpt did not contain any human voice, there were at least two recognizable notes in the excerpt, and the fragment represented as much a possible the maximal timbral diversity (i.e., maximum number of instruments) of the song. In addition, the excerpt was preferably taken from a repeated section of the song. A table with all song titles, artists, and the corresponding genre is given in Table 1 of the Appendix (Supplementary Materials).

Excerpts were extracted directly from .wav files taken from the original CD recordings and stored at an audio sampling rate of 44.1 kHz. For the computation of audio features, all clips were converted to mono by summing both stereo channels. For the two experiments, excerpts of lengths 400 ms (Sample I) and 800 ms (Sample II) were used, extracted from different locations in the song, to which a 20 ms fade-in and fade-out was added. We needed to work with different stimulus durations in Samples I and II because in the original sound similarity sorting task (Müllensiefen et al., 2014), genre was used as a proxy for sound similarity, based on the fact that songs that belong to the same genre are often characterized by similarities in sound (e.g., see Rentfrow et al., 2011). In the absence of a perceptual-computational model of sound similarity at the stage of designing the experimental task, genre was the best proxy available to select groups of songs that would sound similar and at the same time different from other groups of songs, thus allowing to tentatively score the performance on the sorting task of each participant. But from the analysis of the behavioral data obtained for the 400ms excerpt set it became clear that many participants scored close to chance level. After piloting different clip lengths, a duration of 800ms then seemed to produce a distribution of performance scores that better allowed to characterize inter-individual differences.

### 2.3. Experimental procedure

The experimental paradigm was similar to the one used by Gingras et al. (2011) and Giordano et al. (2010). The participants' task was to listen to 16 short excerpts and to sort them into four groups of four items each by their similarity in sound. We deliberately avoided the term “genre” in the instructions and did not specify the nature of the sound similarity. Excerpts were identified by icons on a computer screen, while groups corresponded to boxes. Participants could listen to an excerpt by hovering over its icon, and could move icons around by clicking and dragging. Participants were allowed to listen to each clip as many times as they wished and change their sorting solution as often as necessary. There was no time constraint for the task and participants submitted their sorting solution when they felt that it could not be amended further. Only the final sorted state was recorded and used for subsequent analysis.

### 2.4. Data characteristics

**Figure 1:**
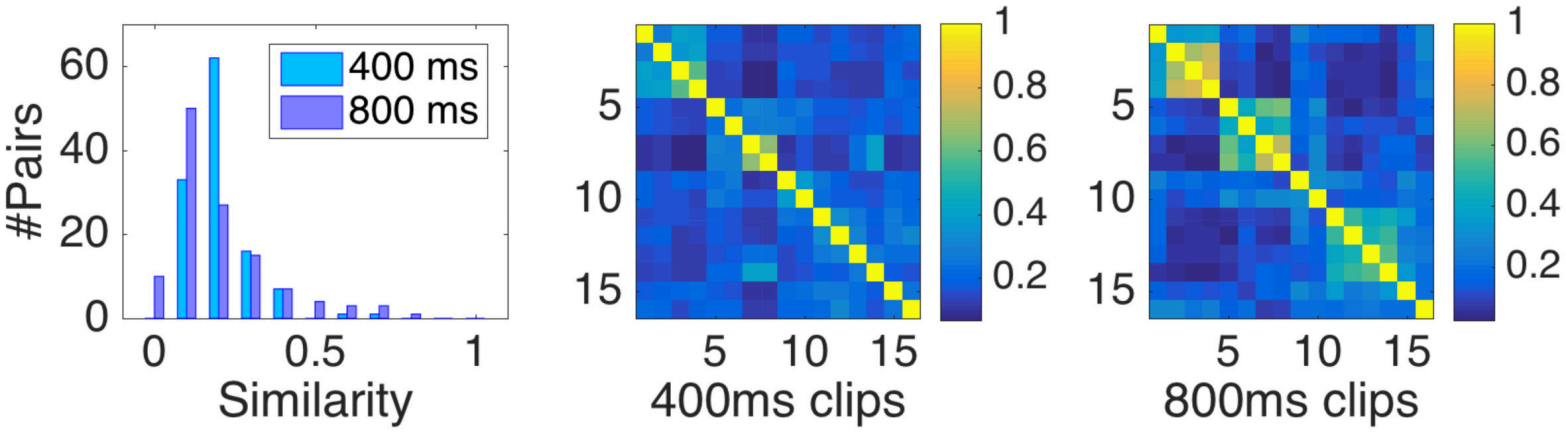
**(Left panel)** Distribution of similarity data, here defined as the relative number of shared classifications of two clips. (**Middle and right panel)** pairwise similarities for Samples I (400 ms) and Sample II (800 ms).

Pairwise perceptual similarity was defined as the relative number of times two clips were placed in the same group by participants. This measure is obtained by dividing the absolute number of times two clips were placed in the same group by the respective number of participants in each sample. The corresponding distribution of similarities with range between zero and one is shown in Figure 1 (left panel).

Recall from Section 2.1 that the demographics of the participant populations from Samples I and II were not matched. In order to rule out potentially confounding effects of demographics on the similarity data, we drew subsamples of Sample I that better matched the demographics of the college-student population of Sample II. Among the 137,399 participants, there were 32,329 participants specifically with age between 18 and 24 years. Thereof 18,639 participants stated “At university” as occupational status, 3,199 participants stated “Education/Training” as their occupation, and 1,957 participants belonged to both categories. However, Pearson correlations between the similarities derived from these subsamples and the set of all participants were very strong, all *r*(118) > 0.992 (*p* < 0.001), which speaks against a pertinent influence of demographics.

Note that the diagonal entries of the similarity matrices depicted in Figure 1 (two rightmost panels) play a distinct role. In fact, they derive from representing the data in matrix form and not from participants' direct classifications themselves (who only encountered distinct clips). The value of the diagonal entries of the matrix automatically equals one, regardless of participants' responses (because every clip trivially shares its own group). However, their inclusion in the model bears the danger of inflating figures of merit such as the coefficient of determination *R*^2^. Because by simply differentiating identical and non-identical clips with a binary variable, one can readily obtain highly significant fits with the similarity data. For that reason, we took a conservative stance and only considered non-identical pairs for the following modeling, corresponding to the lower triangular dissimilarity matrix without diagonal entries (accordingly, the distribution of similarities depicted in the left panel of Figure 1 only represents non-identical pairs). This makes the interpretation of *R*^2^ coefficients more meaningful, but also reduced their magnitude by more than 20% points on average.

## 3. Model structure

**Figure 2:**
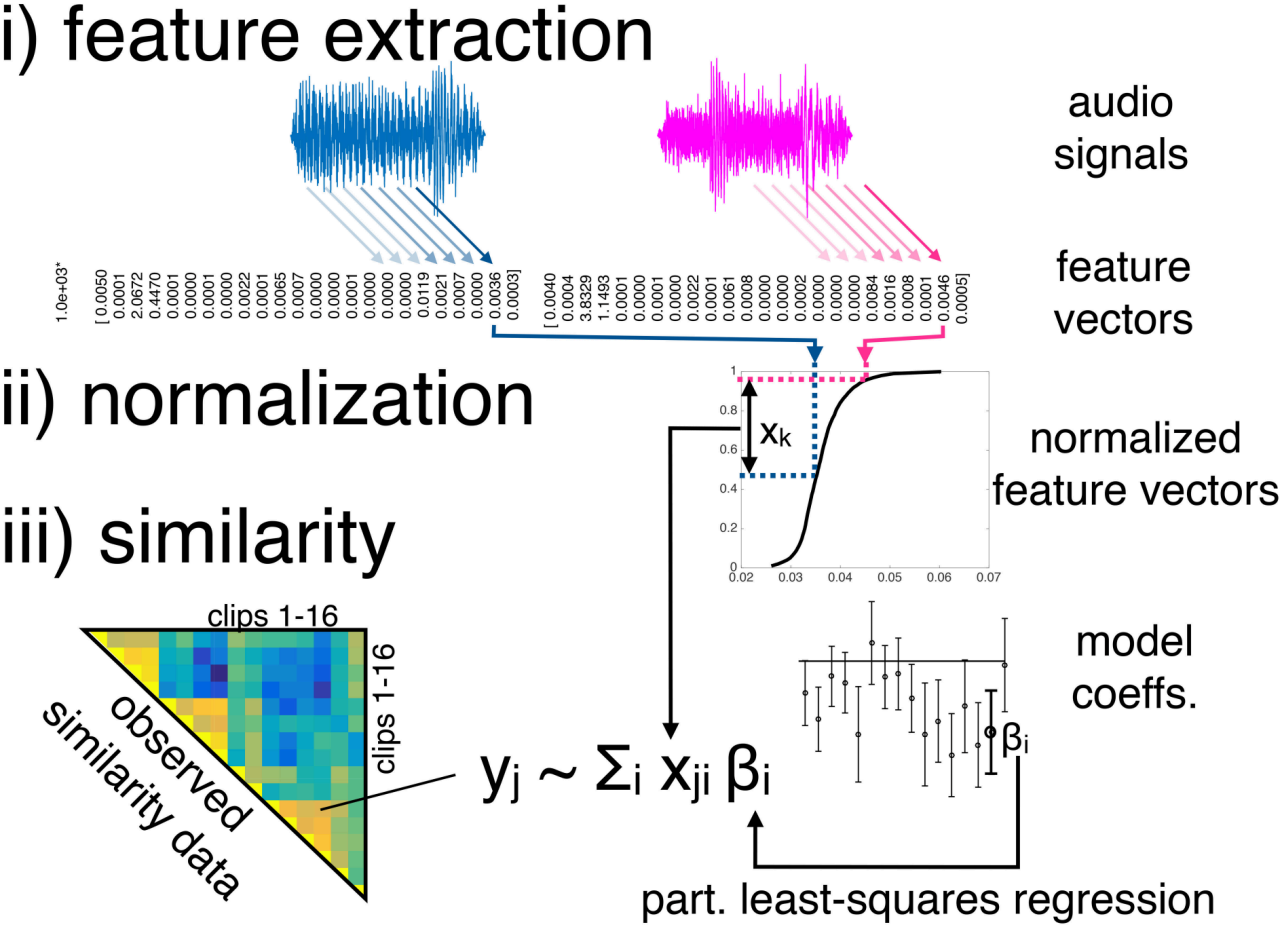
**Basic model structure**. Note that every feature dimension is processed separately before being joined in the regression model.

Modeling the similarity data comprised three main stages: (i) feature extraction from the audio clips, (ii) feature normalization, and the (iii) modeling of pairwise similarities of features. More specifically, we used two sets of audio features, both of which contained 24 features. Both sets were normalized in five different ways (but the normalized features were not pooled). The resulting pairwise distances of clips' audio features were then used as predictor variables in a latent-variable linear regression technique, namely partial least-squares regression (PLSR). Specifically, PLSR attempts to find the multidimensional direction (i.e., the latent variables) in the space of the predictor variables that best explains the maximal variance of the dependent variables. Figure 2 visualizes the three modeling stages. The basic model structure is similar to the timbre dissimilarity model presented by Siedenburg et al. (2016b), but complements stage i) with an additional set of features, considers an array of normalization schemes in stage ii), and applies the model to the case of short music clips instead of isolated instrument tones.

### 3.1. Feature sets

The two feature sets were: (i) a set of 24 timbre descriptors and (ii) 12 Mel-frequency cepstral coefficients (MFCCs) as well as 12 of their Δ-coefficients. In addition we also combined both sets to obtain a third feature set (iii) with 48 features.

#### 3.1.1. Timbre descriptors

We used the Timbre Toolbox (v1.2, Peeters et al., 2011), a large set of audio descriptors that describes the acoustic structure of audio signals with a focus on timbre. For the current purpose, we selected 24 out of its 164 descriptors. This selection possessed great overlap with the 34 descriptors used in Siedenburg et al. (2016b), which had provided a fairly robust model of musical timbre dissimilarity of isolated musical tones, each played individually on instruments of the Western orchestra. In contrast to the isolated tone case, however, ten of the twelve temporal descriptors were not taken into account for the description of clips, because it could be assumed that measures of attack or release-duration would not differ in any meaningful way across the currently used clips, given that they were extracted from the midsts of songs and contained dense musical textures.

Spectral shape descriptors were computed from an ERB-spaced Gammatone filterbank decomposition of the signal. They were measured with (fairly common) settings of 25 ms time frames with 1/2 overlap and summarized via the median and interquartile range as measures of central tendency and variability, respectively. Spectral descriptors included the first four moments of the spectral distribution, such as the spectral centroid that has been shown to correlate with perceived brightness (McAdams, 2013). Additional descriptors of the spectral distribution such as the decrease and flatness were also included, measuring spectral slope with an emphasis on lower frequencies and the peakiness of the spectrum, respectively, but also measures of spectrotemporal variation, relevant to capture spectrotemporal variability (the so-called spectral *flux*) (McAdams et al., 1995). We included four descriptors that were based on the time domain representation of the signal: the frequency and amplitude of energy modulation over time, and the median and interquartile range of the zero crossing rate. A full list of the descriptors is given in Table 2 in the Appendix (Supplementary Materials).

#### 3.1.2. Mel-frequency cepstral coefficients

As an alternative set of features, we considered the commonly-used Mel-frequency cepstral coefficients (MFCCs, Eronen, 2001) and their temporal derivatives. MFCCs are derived via a discrete cosine transform of the log-transformed power of Mel spectra. MFCCs thus represent the shape of an audio signal's spectral envelope: going up from lower to higher coefficients, MFCCs encode increasingly finer scales of spectral detail. MFCCs are standard in various tasks in audio content analysis and music information retrieval and have also been proposed as descriptors for timbre perception (see the review in Siedenburg et al., 2016a). In the current study, we used the first 12 MFCCs and their corresponding 12 ΔMFCCs, i.e., their first derivative over time. Both were computed for 25 ms time frames (1/2 overlap) of the audio signal, and the resulting time series was summarized by the median. These features were provided by the MIRtoolbox (v1.6.1, Lartillot and Toiviainen, 2007).

### 3.2. Feature normalization

In order to regularize the often idiosyncratic distributions of the raw feature values, five normalization schemes were considered:

N1) None (i.e., using raw feature values),
N2) Range normalization to [0, 1],
N3) Z-scores with zero mean and unit standard deviation,
N4) Rank transformation according to the test set: replacing a feature value by the fraction *l*/*L*, with *l* being the feature value's rank within the test set of size *L*,
N5) Rank transformation according to a corpus: replacing a feature value by the fraction *l*′/*L*′, with *l*′ being the feature value's rank within the corpus of size *L*′.

The corpus was obtained by extracting clips from a freely-available audio data set sampled at 44.1 kHz (Homburg et al., 2005). We selected 110 songs for each of the four meta-genres of the current test set (jazz, rock, pop, hiphop), from which we extracted ten 800 ms clips each. The resulting 4,400 clips constituted our corpus. All of the above mentioned features were extracted from each clip of the corpus and used for the corpus-based ranking.

### 3.3. Similarity modeling via partial least-squares regression

Per clip, each feature provided one scalar value. For any pair of clips, feature-wise distances were obtained by taking the absolute difference of the pair's respective feature values. These distances were summarized in a design matrix *X* of size *m* × *n*, where *m* = 120 = 16·15/2 denotes the number of pairs, and *n* denotes the number of features. As outlined above, in a first step we used three sets with (i) *n* = 24 timbre features (from the Timbre Toolbox, TT), (ii) *n* = 24 (Δ)MFCCs, and (iii) *n* = 48 features in the combined set.

In order to handle collinearity of predictors (Peeters et al., 2011), we used partial least-squares regression (PLSR, Geladi and Kowalski, 1986; Wold et al., 2001). PLSR is a regression technique that projects the predicted and observed variables onto respective sets of latent variables, such that the resulting variables' mutual covariance is maximized. More precisely, given a dependent variable *y* and an design matrix *X*, PLSR generates a latent decomposition such that *X* = *TP*′+*E* and *y* = *Uq*′+*F* with loadings matrices *P* (*n* × *k*) and *q* (1 × *k*), and components (“scores”) *T* (*m* × *k*) and U (*m* × *k*) plus error terms *E* and *F*. The matrix W^*^ (*n* × *k*) comprises the predictors' weights, such that *T* = XW^*^. The regression coefficients for the original design matrix can be obtained by β = W^*^q′ (cf., Wold et al., 2001), which yields a link to the generic multiple linear regression (MLR) design via *y* = *Xβ*+*F*. The decomposition maximizes the covariance of *T* and *U*, which yields latent variables that are optimized to capture the linear relation between observations and predictions. In this sense, PLSR also differs from principal component analysis (PCA) followed by MLR, as for instance used by Alluri et al. (2012), since PCA does not specifically adapt the latent decomposition to the dependent variable of interest.

In order to prevent overfitting of the response variable, the model complexity *k* can be selected via cross-validation. We used a model with *k* = 2 latent components, which yielded minimal 8-fold cross-validation errors in a majority of the model and evaluation conditions. We used the implementation provided by the plsregress.m function as part of MATLAB version R2015b (The MathWorks, Inc., Natick, MA), which applies the SIMPLS algorithm (De Jong, 1993).

The importance of individual predictors in the PLSR model was assessed by bootstrapping, which eventually allowed us to construct sparse regression models. For each of the two training conditions, the significance of the individual model coefficients β_*i*_ (*i* = 1, …, *n*) was estimated by bootstrapping the 95% confidence interval of the coefficients (Efron and Tibshirani, 1994; Mehmood et al., 2012). We used a percentile-type method, that is, from the 16 clips per stimulus set, the similarity data of four randomly selected clips (drawn with replacement) were left out from the sample (yielding on average around 60% of the data points intact). This process was repeated 1,000 times. For every coefficient β_*i*_ the resulting 0.025 and 0.975 percentiles were taken as confidence boundaries. If confidence intervals did not overlap with zero, a predictor's contribution was considered to be significant, and the respective feature was selected for the sparse model.

## 4. Model evaluation

The goal of the subsequent model evaluation was to identify from among the three different feature sets and the five different normalization schemes an accurate and robust model of the perceptual similarity data. We place a special focus not only on the question how accurately a statistical model can be fitted to training data, but also on how well the model generalizes to a new set of perceptual data gathered from a different set of audio excerpts. This question is addressed by including sparse models in the subsequent evaluations that are known to generalize better to new datasets (Friedman et al., 2009) and by permuting the data from Sample I and Sample II as training and testsets. This means, each model is both fitted and tested on the datasets from Sample I (400 ms clips) and Sample II (800 ms clips). This results in 2 × 2 evaluation conditions per model. This evaluation setup also allows us to investigate the question how well a model describes the data set it was fitted to and to what degree it might be overfitted to the training data.

The evaluation proceeds in four steps. We first present results for the three feature sets in combination with all five normalization conditions. Secondly, we select a subset of the most relevant features from each model via bootstrapping and recompute the performance of the resulting sparse models. Thirdly, we consider the role of meta information such as genre and the release date of recordings. Finally, we discuss the role of individual acoustic features.

### 4.1. Results: the effect of feature sets and normalization schemes

Table 1 presents the squared Pearson correlation coefficients *R*^2^ for the three full feature sets and five normalization schemes, corresponding to the proportion of variance shared between the model predictions and empirical observations. The results indicate that the perceptual similarities of the 400 ms and 800 ms clips were both predicted with fairly similar accuracy. The combination of the two feature sets, TT+MFCC, yielded the highest *R*^2^ values on training sets as could be expected from the larger pool of features to draw from. However, there are obvious differences between model fits derived on the training sets and model generalization to novel test sets, which suggests that all models considered at this point generalize rather poorly to unseen data. In fact, successful generalization is a rare exception with only four out of 30 predictions of unseen data yielding correlations that are significant at the α = 0.01 level. Generalization of models based on MFCCs was particularly poor and did not provide a single significant correlation on a novel test set.

**Table 1:** *****R***^**2**^ coefficients as performance indicators for full models derived from combining five normalization schemes (N1–N5) and three feature sets (TT, Timbre Toolbox; MFCC, MFCC coefficients and MFCC delta coefficients), each evaluated in the two training and testing conditions from 400 (I) and 800 ms (II) clips**.

			**TEST**									
			**N1**		**N2**		**N3**		**N4**		**N5**	
			**Raw**		**Range**		**z-scores**		***r*****-test**		**r-corpus**	
			**I**	**II**	**I**	**II**	**I**	**II**	**I**	**II**	**I**	**II**
**TRAIN**	TT	I	–	–	0.07	0.08	0.07	0.06	0.33	–	0.11	0.09
		II	–	–	–	0.18	–	0.19	–	0.22	–	0.25
		Mean		–		0.08		0.08		**0.14**		0.11

	MFCC	I	0.08	–	0.21	–	0.20	–	0.21	–	0.23	–
		II	–	0.15	–	0.20	–	0.20	–	0.22	–	0.24
		Mean		0.06		0.10		0.10		0.11		**0.12**

	TT+MFCC	I	–	–	0.25	0.06	0.25	–	0.47	–	0.27	–
		II	–	–	–	0.24	–	0.26	–	0.39	–	0.33
		Mean		–		0.14		0.13		**0.22**		0.15

*Note that R^2^ coefficients < 0.06 that correspond to non-significant correlations at p > 0.01 are not displayed for the sake of clarity. The mean for each combination of normalization scheme and feature set across the four training-test evaluation conditions is given in the last row of each cell (non-significant correlations are considered a zero entry in the computation of the mean). Best average performance per feature set is given in bold font*.

**Figure 3:**
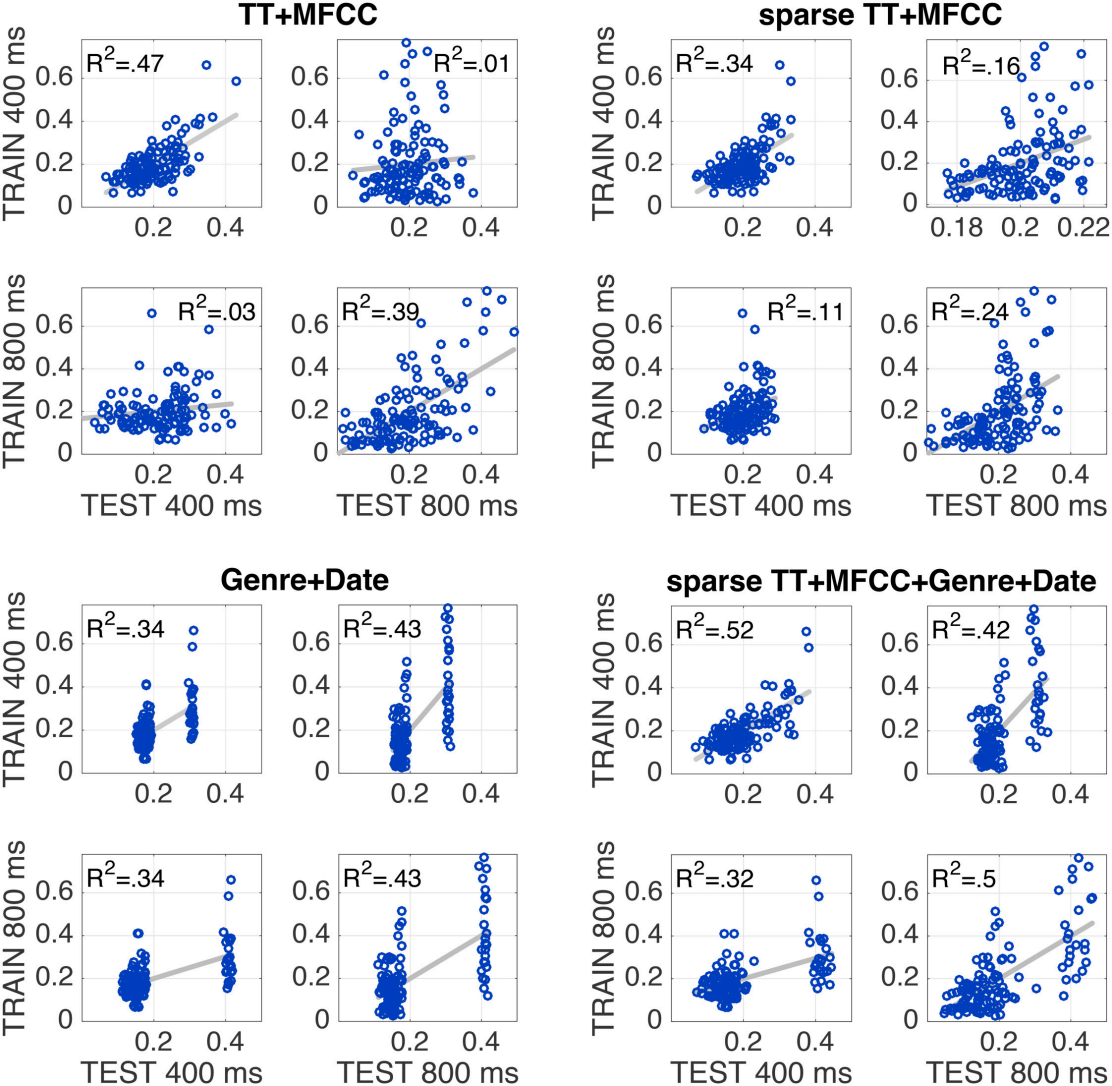
**Every individual plot shows the correspondence between model predictions (x-axis) and empirically observed similarities (y-axis)**. Every panel shows models that were trained to 400 ms (panel top row) or 800 ms clips (panel bottom row), and tested on the same two sets (left vs. right columns). Top left panel shows full feature set; top right: sparse feature selection; bottom left: non-acoustic variables only; bottom right: sparse model together with non-acoustic variables. All models utilize test-ranked features (N4).

In terms of the normalization schemes, models using the test-set-based ranking (N4) produced the highest performance values overall. In particular for the combined feature set TT+MFCC, it yielded the best fit to the training data, potentially indicating that participants rely on relative differences within a specific acoustic context (namely the test set), rather than on absolute differences of acoustic features. Figure 3 (top left panel) shows the scatterplot of the corresponding TT+MFCC (N4) model in all four evaluation conditions, graphically depicting how model fits decrease from when training and test dataset are identical to when datasets for model training and test differ. This decrease in model fit may be interpreted as an indicator of model overfitting. Hence, in the next evaluation step we aim to achieve better generalization performance and avoid overfitting by applying feature selection.

### 4.2. Feature selection

**Figure 4:**
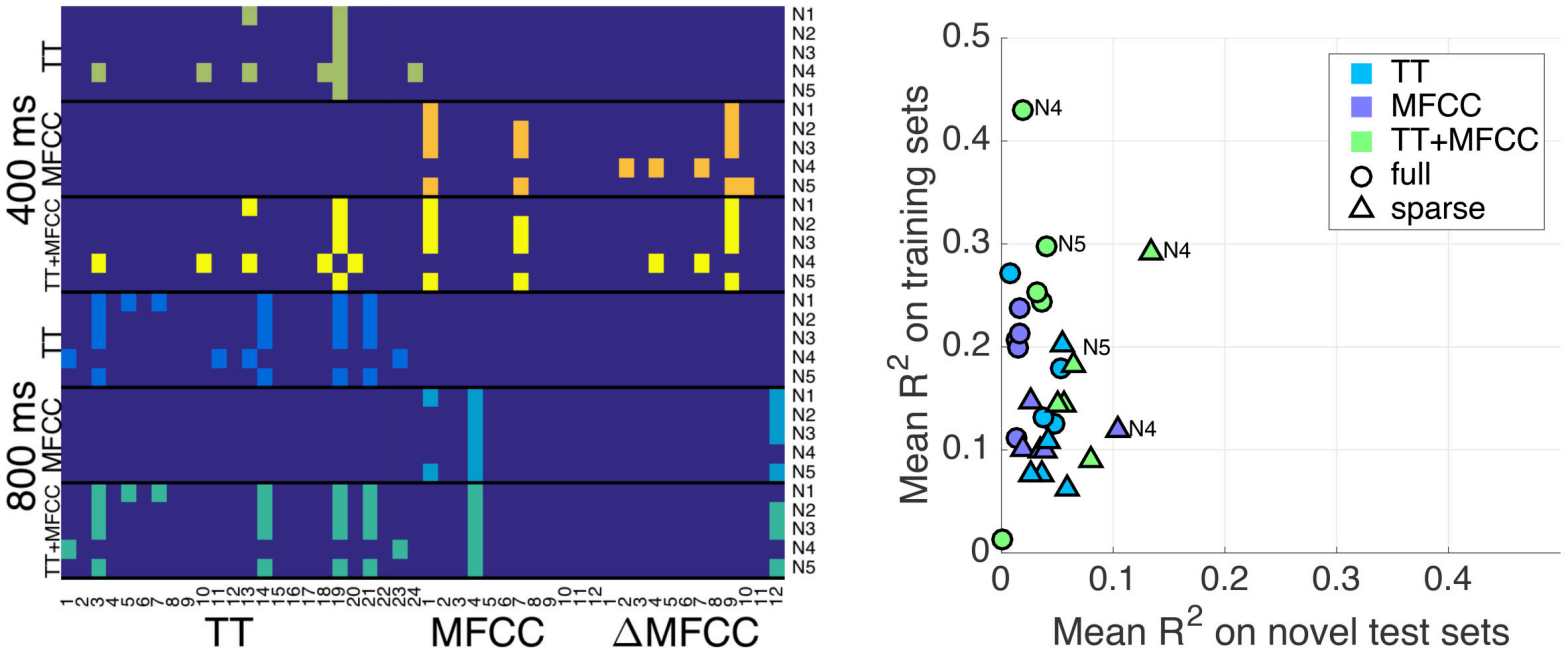
**(Left panel)** Predictors selected for the sparse models. Pixels in colors other than dark blue refer to selected variables. The x-axis shows features, namely TT descriptors (1–24, see Table 2 in Supplementary Materials), and the 12 MFCCs and 12 ΔMFCCs. The y-axis concatenates all conditions including the 400 ms and 800 ms clips and the feature sets TT, MFCC, and TT+MFCC. Normalizations N1–N5 are annotated on the right. **(Right panel)** Overview of results from the solely acoustic models. Plot shows the mean fit to the training set (y-axis) and the mean fit on novel sets (x-axis). Circles correspond to the full models, triangles to sparse sets of features. A few normalizations are annotated.

We applied the feature selection approach described in Section 3.3 to obtain sparse models. This naturally led to different configurations of significant predictors per model and evaluation condition, which are displayed in Figure 4. The plot shows that the selection was fairly consistent across the different feature sets, in the sense that the combined feature set TT+MFCC roughly comprised the features already selected for the individual sets TT and MFCC. For the 400 ms clips, an average of 2.2, 3.0, and 4.6 significant variables were retained for the TT, MFCC, and TT+MFCC features sets, respectively (averaged across the five normalizations). For 800 ms clips, an average number of 4.4, 2.2, and 5.6 features were retained for the three respective feature sets. However, note that the set of features selected for the 400 and 800 ms clips is quite different. In particular for the test-ranked normalization (N4), the two sets do not share any common member.

Table 2 shows the results in all conditions for the sparse models^2^. There are 14/30 significant correlations for unseen test data, which is an improvement compared to the full models (4/30), yet still surprisingly low overall. The mean performance of sparse models (across all four evaluation conditions) was rather similar to the performance of the full models, which means the increase in generalization performance was traded against a decrease of accuracy on the training sets. The best model was obtained by the combined model TT+MFCC with the test-rank normalization (N4), with an average fit of *R*^2^ = 0.29 on the training data and *R*^2^ = 0.14 on novel test data. The detailed scatterplots of predictions and observations are shown in Figure 3.

**Table 2:** **Performance of sparse models**.

			**TEST**									
			**N1**		**N2**		**N3**		**N4**		**N5**	
			**Raw**		**Range**		**z-scores**		***r*****-test**		**r-corpus**	
			**I**	**II**	**I**	**II**	**I**	**II**	**I**	**II**	**I**	**II**
**TRAIN**	Sparse TT	I	–	0.11	–	0.06	–	–	0.24	–	–	0.07
		II	–	0.11	–	0.14	–	0.14	0.10	0.16	–	0.20
		Mean		0.05		0.05		0.03		**0.12**		0.07

	Sparse MFCC	I	0.08	–	0.09	0.06	0.09	0.06	0.12	0.12	0.11	–
		II	–	0.12	–	0.11	–	0.11	0.09	0.12	–	0.18
		Mean		0.05		0.06		0.07		**0.11**		0.07

	Sparse TT+MFCC	I	0.07	0.11	0.12	0.07	0.12	0.06	0.34	0.16	0.13	0.08
		II	–	0.11	–	0.16	–	0.16	0.11	0.24	–	0.24
		Mean		0.07		0.09		0.09		**0.21**		0.11

*R^2^ coefficients are shown for the five normalizations (N1–N5) and three feature sets, each evaluated in the two training and testing conditions from 400 (I) and 800 ms (II) clips. Correlations with p > 0.01(R^2^ < 0.06) are not displayed for the sake of clarity. Condition means are concatenated below. Best average performance per feature set is given in bold font*.

Figure 4 (right panel) gives an overview and summary of the behavior of all models considered so far in terms of accuracy and generalization capacities: the x-axis displays the mean *R*^2^ coefficients on novel test sets (i.e., the average of the off-diagonal values of the previously presented tables) and the y-axis represents the fit on the training sets (i.e., average of diagonal values). Generally, we are interested in a reasonable trade-off between both measures, which currently only appears to be achieved by the sparse TT+MFCC models with test-rank normalization (N4). In this sense, the figure illustrates two important methodological results: (a) The combined feature set TT+MFCC is superior to both TT and MFCC, (b) sparse variable selection is a means to trade accuracy on the training set against a greater ability of the models to generalize to unseen datasets.

### 4.3. The role of genre and release date

In a final step, we included non-acoustic information as predictor variables that were taken from the meta-data of the clips. Specifically, we considered the categorical variable of genre as well as the songs' release dates. However, is it important to keep in mind that the concept of genre is notoriously ambiguous (Craft et al., 2007). In the current case, genre was correlated not only with the release date of recordings, but also with recording techniques, instrumentation, and thus also with qualitative timbral similarity modeled by the continuously varying audio features utilized here. Therefore, this step was of exploratory nature and attempted to set the prediction results of the acoustic model into relation with approaches relying on meta information.

Genre was coded as binary predictor *G* indicating whether two clips shared the same genre (G = 0) or not (G = 1). As Figure 3 (bottom right panel) demonstrates, adding these two predictors to the model with the best generalization performance, the sparse test-ranked-normalized TT+MFCC model, yielded a substantial increase in model performance of at least 18 percentage points in *R*^2^. At the same time, the model that solely utilizes meta information (Genre+Date) robustly partitions the underlying pairs into fairly similar vs. dissimilar pairs. The computational analyses presented in the present paper thus confirm the efficiency of genre as a proxy for the selection of stimuli (as described in Section 2.2), with genre explaining the vast majority of the variability in the behavioral data. Moreover, the Genre+Date model here yielded better *R*^2^ values in generalization than the model that relies on both acoustic and meta variables. The latter, indeed surprising finding could be taken as evidence for that listeners from Sample I and II used different weightings of acoustic information in their responses, potentially due to the different lengths of excerpts.

### 4.4. Role of individual features

**Figure 5:**
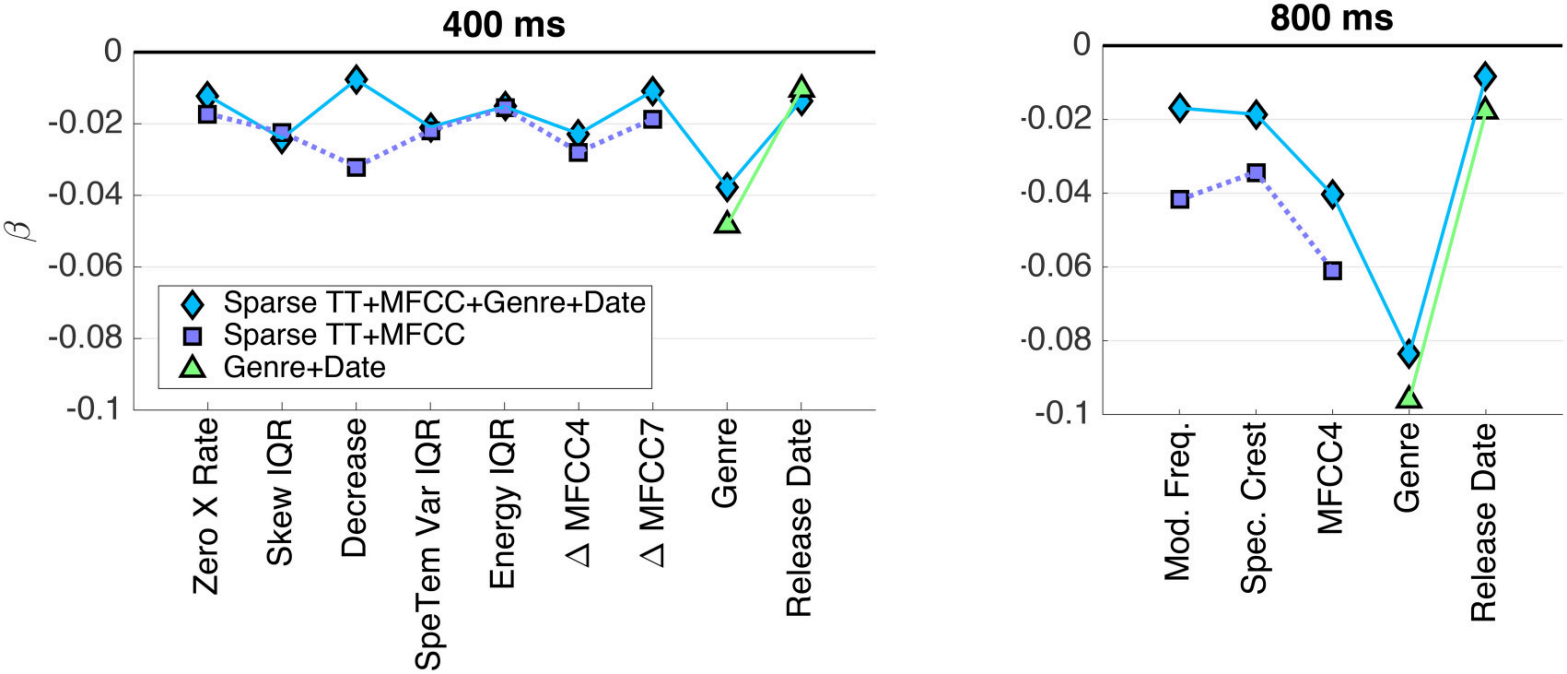
**PLSR coefficients β for the sparse model with test-rank normalization (N4), coefficients of the same model including genre and release date, as well as genre and release only**. **(Left panel)** 400 ms clips; **(right panel)** 800 ms clips.

By virtue of the parsimony of the sparse models, it is possible to take a more detailed look at the individual weightings of predictor variables. Here, we consider the exemplary case of the TT+MFCC (+Genre+Date) models with test-set ranking (N4). Figure 5 shows the (standardized) regression coefficients β, which reflect the relative importance of the individual predictors for the prediction of similarity (*y* = *Xβ* + *F*).

For the models that included both acoustic and meta descriptors, the plots indicate that the genre descriptor was the most heavily weighted variable for both stimulus sets, and the date of release showed a by far smaller influence. Note that we found a very similar relation between the effect sizes of both variables for the model solely using genre and date information (the coefficients of which are not shown here). Regarding acoustic descriptors, the selections for both stimulus sets represent both spectral and spectrotemporal information: the spectral envelope distribution is represented by features such as Crest, Decrease, and MFCCs, whereas (spectro-)temporal modulations are represented by Modulation Frequency, Spectrotemporal Variation, and Δ MFCCs. Specifically, MFCC no. 4 was by a large margin the most important acoustic feature for the 800 ms clips, whereas Δ MFCC no. 4 was among the most important ones for the 400 ms clips.

From a more general stance, the presented evaluation, using five normalization conditions and three acoustic feature sets, indicates that one should not overestimate the universality of distinct acoustic features. In fact, the best model configuration did not share any features across the two stimulus sets. A plausible hypothesis could be that the duration of clips plays a pertinent role in the ways in which listeners compile and weight acoustic information from short music clips.

## 5. Discussion

### 5.1. Summary

The main aim of this study was the development of the first audio-feature-based model for the prediction of human sound similarity judgements of short audio excerpts. We used partial least-squares regression in order to map from acoustic to perceptual similarity. Before entering the regression model, acoustic dissimilarities were normalized by using five schemes: (N1) raw feature values, (N2) range-normalization, (N3) z-scores, (N4) rank-transformation according to test set, and (N5) rank transformation according to a corpus. We then followed an exhaustive combinatorial approach that combined these five normalization schemes with two important candidate feature sets, the Timbre Toolbox (Peeters et al., 2011) and MFCCs, each of which contributed with 24 audio features. Importantly, each candidate model was assessed on the dataset it was fitted to, as well as on a set of novel audio excerpts. Our results indicate that combining both feature sets resulted in the most powerful model, in particular when being used with a test-set based rank transformation (N4). And even the sparse models with their drastically reduced numbers of features generally contained members from both features sets. This speaks for the complementary nature of Timbre Toolbox descriptors and MFCCs when it comes to the description of the similarity of music clips.

In line with the well-documented behavior of sparse models in terms of better generalization (e.g., Friedman et al., 2009, Ch. 16.2.2), we also found a trade-off between model performance on the training set vs. the models' enhanced ability to generalize to a new dataset. The best performing sparse model achieves an *R*^2^ of up to 0.34 when evaluated on the dataset is was trained on and an *R*^2^ of up to 0.16 when evaluated on a new dataset. This result for the first time provides evidence that a significant portion of the variance in the similarity perception of short music clips can be explained by acoustic features related to timbre. The fact that including only two variables encoding meta-information, and most importantly musical genre, substantially increased model performance (up to *R*^2^ values of 0.52) suggests that the models based on acoustic features do not capture all information that participants are able to extract from the short audio clips and use for the similarity grouping. This finding also implies that great care should be taken in order to control for the effects of variables such as genre in future studies of sound and music similarity.

This last result is analogous to the importance of categorical information in the timbral dissimilarity of isolated instrument tones reported by Siedenburg et al. (2016b), where the addition of sound-source and instrument-family-related variables to a model based on acoustic features significantly improved the prediction of dissimilarity ratings (also see, Lemaitre et al., 2010). In this respect, the current results suggest that even if instructed to focus on low level auditory features (i.e., “the sound”), participants' responses are affected by higher level concepts such as genre. Although differences in timbral qualities, here measured by continuously varying audio features, likely constitute an important part of genre, genre categories might also be inferred from higher-level stylistic musical features such as rudimentary rhythmic or pitch-related information that are still discernible in some of the clips. The current results then suggest that the inference of higher level concepts such as emotion or genre from short audio clips is based on more than timbral qualities, but rather on a complex mixture of acoustical, musical, and categorical (or higher-level) types of features. Notably, the exact weightings of these variables may vary with the duration of the excerpts. From the opposite perspective, the modeling infrastructure built up here could of course be applied to exploring the acoustic features utilized by humans in explicit genre identification tasks.

### 5.2. Limitations of the current study and future perspectives

This study represents the first rigorous attempt to build quantitative models that describe the perception of short audio excerpts based on audio feature extraction. Whereas, we have achieved encouraging accuracies on the training data, there is clearly room for improvement in future studies, in particular when it comes to generalization performance. A limitation of the current study is the fact that the two datasets differed in terms of the length of the excerpts (400 vs. 800 ms) whereas the modeling approach assumed that the same features are equally suitable for clips of both lengths. But this assumption might not be necessarily true. Hence, a future replication of this study should include different datasets with clips of the same lengths. Potentially, this might also help to achieve better generalization results. Specifically, it would be necessary to confirm the performance accuracy of the model with the best generalization performance (i.e., the sparse version of the TT+MFCC feature set using test-set-based rank normalization plus meta information) on a completely new dataset. New audio excerpts could be selected from a corpus according to their similarity predicted by the model, allowing us to generate precise hypotheses about the number of times the new excerpts are grouped together in the grouping paradigm. Using fully randomized approaches for determining the clips' starting points in the song, as proposed by Thiesen et al. (2016), would likely add further robustness to the experimental design.

It is also worth noting that the similarity data used in this study were derived from a grouping paradigm that required participants to make categorical decisions and it is unclear whether this specific paradigm introduced any sort of bias into the data. However, several other experimental paradigms can be used to obtain similarity data from participants and might be employed in future studies alongside the grouping paradigm (Giordano et al., 2011). These include pairwise similarity ratings on fine-grained scales, rankings of clips in relation to an anchor stimulus, triadic comparisons (Allan et al., 2007) or similarity comparisons of two pairs of clips.

In order to capture relevant additional information contained in the audio clips beyond timbral features, future investigations could also include mid-level features that describe aspects of the rhythmic, harmomic and pitch patterns (e.g., Müller, 2015). However, a systematic study of the participants' strategies used for arriving at perceptual categorizations of short audio clips would be a most helpful starting point for selecting features for subsequent modeling. The *thinking-aloud method* (Kuusela and Paul, 2000) commonly used in HCI research and other areas could be highly instrumental here to obtain qualitative insights into the cognitive processes employed when perceiving short audio clips.

Eventually, a reliable computational model of the perceptual similarity of short audio clips can serve as the basis for a refined individual differences test that assesses the ability to make fine-grained distinction between short musical excerpts. A computational model is necessary in order to create a test that is adaptive and homes in on the individual participant's ability level for judging sound similarities. In the case of the grouping paradigm, the computational model would be used for automatically selecting sets of clips that are easy vs. difficult to group, i.e., that differ in their within/between-group similarity. But the scientific value of a test that tracks and predicts an individual's ability to make similarity judgements lies not only in potential use as a new testing tool. Significant additional value comes from the cognitive insights gained from applying music information retrieval techniques to model complex perceptual processes.

## Author contributions

Both authors KS and DM contributed equally to the design of the data modeling and writing of the manuscript. KS was mainly responsible for the acoustic feature models and data analysis. DM was responsible for data collection at Goldsmiths, University of London.

### Conflict of interest statement

The authors declare that the research was conducted in the absence of any commercial or financial relationships that could be construed as a potential conflict of interest.

## Footnotes

^1^Because participants of Sample II were aggregated from several individual experiments, unfortunately it was impossible at this stage to track participants' individual demographic information.

^2^The number of PLSR latent components *k* was naturally reduced to *k* = 1, if only one feature was selected by bootstrapping in the respective condition.
