# Supplementary material for: Modeling Timbre Similarity of Short Music Clips
Source: Front Psychol. 2017 Apr 26;8:639. doi: 10.3389/fpsyg.2017.00639 (PMC5405345; doi:10.3389/fpsyg.2017.00639)
Supplement: Supplementary file 1 [file Table1.pdf]

1 LIST OF SONGS AND ARTISTS

Table 1. List of song titles from which clips were extracted, including the corresponding artist names and genres.

	Song title	Artist	Genre	Release date
1)	*Azucar*	Eddie Palmieri	Jazz	1965
2)	*Evolution*	Roy Ayers	Jazz	1975
3)	*Speak No Evil*	Wayne Shorter	Jazz	1966
4)	*Work Song*	Nat Adderley	Jazz	1960
5)	*Crazy on You*	Heart	Rock	1976
6)	*Oh Atlanta*	Little Feat	Rock	1974
7)	*Rock 'n Roll Fantasy*	Bad Company	Rock	1979
8)	*Talk to Ya Later*	The Tubes	Rock	1981
9)	*Carnival*	The Cardigans	Pop	1995
10)	*I Wanna Love You Forever*	Jessica Simpson	Pop	1999
11)	*So Real*	Mandy Moore	Pop	1999
12)	*The Sign*	Ace of Base	Pop	1993
13)	*By The Time I Get To Arizona*	Public Enemy	Hip hop	1991
14)	*We Aint Goin Out Like That*	Cypress Hill	Hip hop	1993
15)	*Still Not a Player*	Big Punisher	Hip hop	1998
16)	*Who's That Girl?*	Eve	Hip hop	2001

2 LIST OF TIMBRE TOOLBOX DESCRIPTORS

Table 2. List of acoustic descriptors from the Timbre Toolbox (Peeters et al., 2011). Medians (med) and interquartile range (IQR) summarize the time-varying descriptors computed over time frames of 25 ms. Square brackets provide descriptor units (a: audio signal amplitude, F: ERB-rate units). Temporal descriptors are computed from the signal energy (temporal) envelope, spectral (and spectro-temporal) descriptors from the ERB gammatone filterbank representation.

Name of audio descriptor
1) Frequency of energy modulation [Hz]
2) Amplitude of energy modulation [a]
3) Zero crossing rate (med) [-]
4) Zero crossing rate (iqr) [-]
5) Centroid (med) [F]
6) Centroid (IQR) [F]
7) Spread (med) [F]
8) Spread (IQR) [F]
9) Skew (med) [-]
10) Skew (IQR) [-]
11) Kurtosis (med) -
12) Kurtosis (IQR) [-]
13) Decrease (med) [-]
14) Decrease (IQR) [-]
15) Rolloff (med) [F]
16) Rolloff (IQR) [F]
17) Spectro-temporal variation (med) [-]
18) Spectro-temporal variation (IQR) [-]
19) Frame energy (med) [a^2]
20) Frame energy (IQR) [a^2]
21) Flatness (med) [-]
22) Flatness (IQR) [-]
23) Crest (med) [-]
24) Crest (IQR) [-]
